# Statistical inference in ensemble modeling of cellular metabolism

**DOI:** 10.1371/journal.pcbi.1007536

**Published:** 2019-12-09

**Authors:** Tuure Hameri, Marc-Olivier Boldi, Vassily Hatzimanikatis

**Affiliations:** 1 Laboratory of Computational Systems Biotechnology (LCSB), Swiss Federal Institute of Technology (EPFL), Lausanne, Switzerland; 2 Department of Operations, Faculty of Business and Economics, Anthropole, University of Lausanne, Lausanne, Switzerland; CPERI, GREECE

## Abstract

Kinetic models of metabolism can be constructed to predict cellular regulation and devise metabolic engineering strategies, and various promising computational workflows have been developed in recent years for this. Due to the uncertainty in the kinetic parameter values required to build kinetic models, these workflows rely on ensemble modeling (EM) principles for sampling and building populations of models describing observed physiologies. Sensitivity coefficients from metabolic control analysis (MCA) of kinetic models can provide important insight about cellular control around a given physiological steady state. However, despite considering populations of kinetic models and their model outputs, current approaches do not provide adequate tools for statistical inference. To derive conclusions from model outputs, such as MCA sensitivity coefficients, it is necessary to rank/compare populations of variables with each other. Currently existing workflows consider confidence intervals (CIs) that are derived independently for each comparable variable. Hence, it is important to derive simultaneous CIs for the variables that we wish to rank/compare. Herein, we used an existing large-scale kinetic model of *Escherichia Coli* metabolism to present how univariate CIs can lead to incorrect conclusions, and we present a new workflow that applies three different multivariate statistical approaches. We use the Bonferroni and the exact normal methods to build symmetric CIs using the normality assumptions. We then suggest how bootstrapping can compute asymmetric CIs whilst relaxing this normality assumption. We conclude that the Bonferroni and the exact normal methods can provide simple and efficient ways for constructing reliable CIs, with the exact normal method favored over the Bonferroni when the compared variables present dependencies. Bootstrapping, despite its significantly higher computational cost, is recommended when comparing non-normal distributions of variables. Additionally, we show how the Bonferroni method can readily be used to estimate required sample numbers to attain a certain CI size.

## Author summary

Due to various sources of uncertainty, populations of kinetic models of metabolism are generally constructed to derive conclusions about the dynamics of the modeled physiology. However, computational ensemble modeling (EM) frameworks for building populations of kinetic models do not systematically handle the uncertainty underlying model-based conclusions. Although kinetic models could suggest that modifying the level of certain enzymes would on average increase a flux of interest, this information is incomplete if we do not know with what certainty the model predicts this. We can instead use statistical inference approaches to quantify the level of confidence for which certain model conclusions lie within the confidence intervals. We demonstrate how three statistical methodologies can be applied to construct confidence intervals around distributions of output variables derived from populations of kinetic models. We discuss the advantages and disadvantages of applying these three methods and provide advice on their usage in EM. This will lead to improved metabolic engineering decisions by helping engineers focus on the most robust conclusions.

## Introduction

Kinetic models are becoming essential computational tools for studying the metabolism of organisms and for understanding the dynamics of their cellular biochemical interactions [1]. However, the construction of kinetic models remains a challenging endeavor as there are large uncertainties in the rate expressions describing all the reactions making up these cellular interactions [2]. This is often because reaction mechanisms are rarely fully characterized for an organism, making it difficult to select appropriate rate expressions for reactions, and information on the parameter values required by these expressions is very scarce. Several ensemble modeling (EM) approaches that assign kinetic mechanisms to reactions, incorporate experimental data, and sample unknown kinetic parameter values have emerged for generating populations of kinetic models [3–6]. Yet, given the promising methodologies that exist for constructing populations of large-scale kinetic models, the community lacks procedures for examining their uncertainty.

Kinetic models are generally constructed with a particular objective, such as improving a substrate’s production, increasing cellular growth, or advising experimentalists on which physiological properties should be measured [1]. Irrespective of the objective, comparing populations of variables—such as metabolic control analysis (MCA) sensitivity coefficients—computed from the kinetic models is a fundamental step in deriving conclusions from computational modeling and engineering. To meaningfully compare populations of variables, it is important to consider their associated uncertainty. Unfortunately, innumerous statistical approaches exist for this, making it sometimes a dubious task to select the “correct” method [7]. Despite originating from statistical mechanics, EM has only been employed in systems biology for two decades [8], and its use of statistical methods for managing uncertainty remains, to our knowledge, untapped.

Kinetic models of metabolism are generally constructed around a given steady state of interest that characterizes the system. Assuming that we know the metabolite concentrations, the flux values, and the reaction mechanisms describing the system, we still have uncertainty in the kinetic parameter values. The Optimization and Risk Analysis of Complex Living Entities (ORACLE) framework handles this uncertainty by considering multiple alternative sets of models by sampling the parameter space until enough models are obtained such that the mean and several other statistical modes of the model outputs converge [4, 9, 10]. Another EM approach generates populations of models to search for a unique model that best fits experimental data to construct a time-course dynamic model that describes the system [3, 11]. A workflow for constructing kinetic models considers populations and assesses statistical significance using the univariate analysis of uncertainty in variables [5]. However, these frameworks for building kinetic models do not appear to consider multivariate statistical methods when accounting for uncertainty.

The purpose of this paper is to suggest how multivariate statistical approaches can be used to construct simultaneous confidence intervals (CIs) for considering uncertainty in the outputs of populations of kinetic models. As there is no unique approach for constructing simultaneous CIs, we addressed this goal by assessing several different methods, which each come with certain underlying assumptions and caveats that should be taken into consideration before application. We compared different approaches that can be applied to our data and make recommendations on how such approaches can serve the community by attributing statistical significance to variables and handling uncertainty.

Simultaneous CIs are ranges that contain the true means of a set of variables with a fixed probability called coverage. Unlike well-known univariate CIs, simultaneous CIs calculate their coverage by accounting for the multiplicity of variables. Their constructions can be approximate or very technical, depending on the underlying distribution of the data. In this paper, we present three multivariate methods: Bonferroni’s correction (BCI), the exact normal (ENCI), and the bootstrap (BootCI). We discuss their advantages, disadvantages, and assumptions to suggest how these methods can be successfully applied for comparing variables computed with EM techniques.

**Fig 1:**
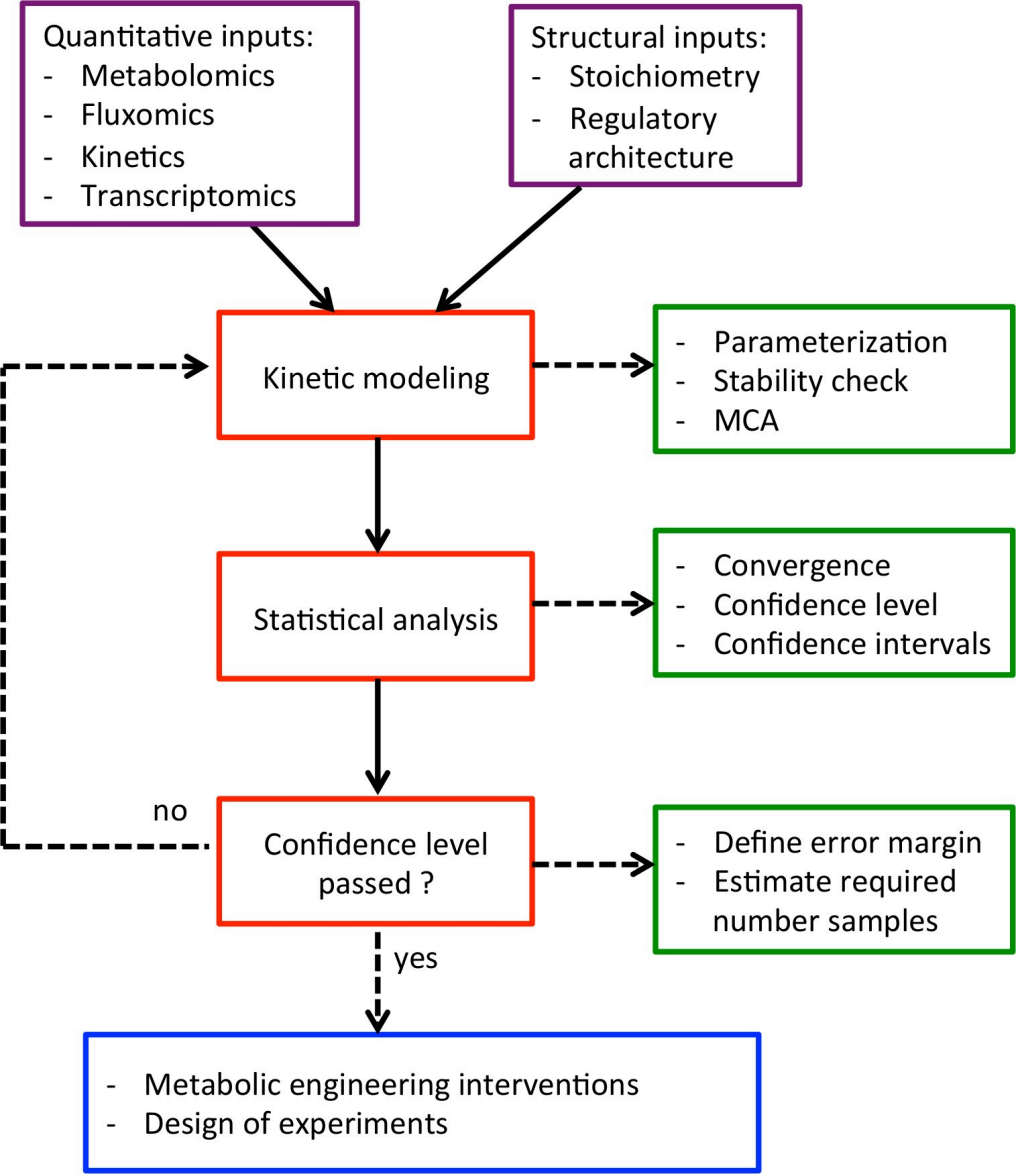
Schematic diagram of workflow carried out in the study. Key steps (red) of the workflow for constructing CIs, with necessary inputs in purple. When the necessary tasks (green) are completed for each step (red), one can move to the next step, repeating the cycle when the confidence intervals do not pass. Only after robust CIs are constructed should one move on to the applications in blue to prevent incorrect conclusions.

To apply these methods, we used a published kinetic model [12] of aerobically grown *E*. *coli* that was derived from the iJO1366 genome-scale reconstruction [13]. The ORACLE framework was used to compute populations of flux control coefficients (FCCs) derived with MCA. The FCCs represent the fold change in a specific flux with respect to the perturbation of an enzyme’s activity of *p* = 275 enzymatic reactions with respect to their enzymes. We studied the FCCs of *n* = 50,000 kinetic models using the three previously mentioned methods for constructing simultaneous CIs and suggested a workflow (Fig 1) for applying them. The algorithms used for constructing the simultaneous CIs are available in the supplementary material (S1 Methods).

## Results and discussion

### Kinetic models studied

Because we needed an ensemble of kinetic models and quantitative model outputs that we could assess statistically, we used a published kinetic model of *E*. *coli* from Hameri et al. [12] for this study. Their reduced stoichiometric model of *E*. *coli* was obtained using the redGEM and lumpGEM algorithms [14, 15] from the iJO1366 genome-scale metabolic model [13]. It is composed of 277 enzymatic reactions and 160 metabolites distributed over the cytosol, periplasm, and extracellular space (S1 Table).

We studied four different operational configurations—also referred to as flux directionality profiles (FDPs) in their publication [12]—that were able to characterize the physiology of aerobically grown *E*. *coli*. These four FDPs each have different reaction directionalities, and a reference steady state was selected for the fluxes and the metabolite concentrations for each one. To account for uncertainty in kinetic parameters, the authors considered populations of kinetic models by efficiently sampling this parameter space with ORACLE. The populations of 50,000 kinetic models from their work [12], were used for each FDP to study the FCCs. As our study focused on comparing statistical methods for deriving CIs around the outputs of populations of kinetic models, we do not discuss the differences in these four operational configurations (FDPs) nor their biological implications and refer the reader instead to the original publication [12].

### Uncertainty in flux control coefficients

FCCs derived from MCA have been used for metabolic engineering purposes to provide insight into the rate limiting steps of a metabolic network. For example, comparing FCCs can help locate which enzyme could be edited to achieve a certain target metabolic state. Hence, we used FCCs as variables to demonstrate how CIs can be constructed around them. For our initial studies into CIs, we focused on the first FDP from Hameri et al. [12] (FDP1) and refer to it herein as Case 1. To illustrate simultaneous CIs in this section, it was easier to focus on Case 1, and the other three alternative cases are presented only in later sections. For this work, we considered the *n* = 50,000 stable kinetic models of Case 1 that they [12] generated with the ORACLE workflow. We consider *p* = 275 FCCs of glucose uptake (GLCptspp) to determine which enzymes have the most control on it and could be of interest for editing. GLCptspp was chosen as an example rather than for any specific biological reasons/objective. The *p* = 275 FCCs will be considered as the variables, for which we have *n* = 50,000 observations (i.e. kinetic models).

### Confidence intervals

**Fig 2:**
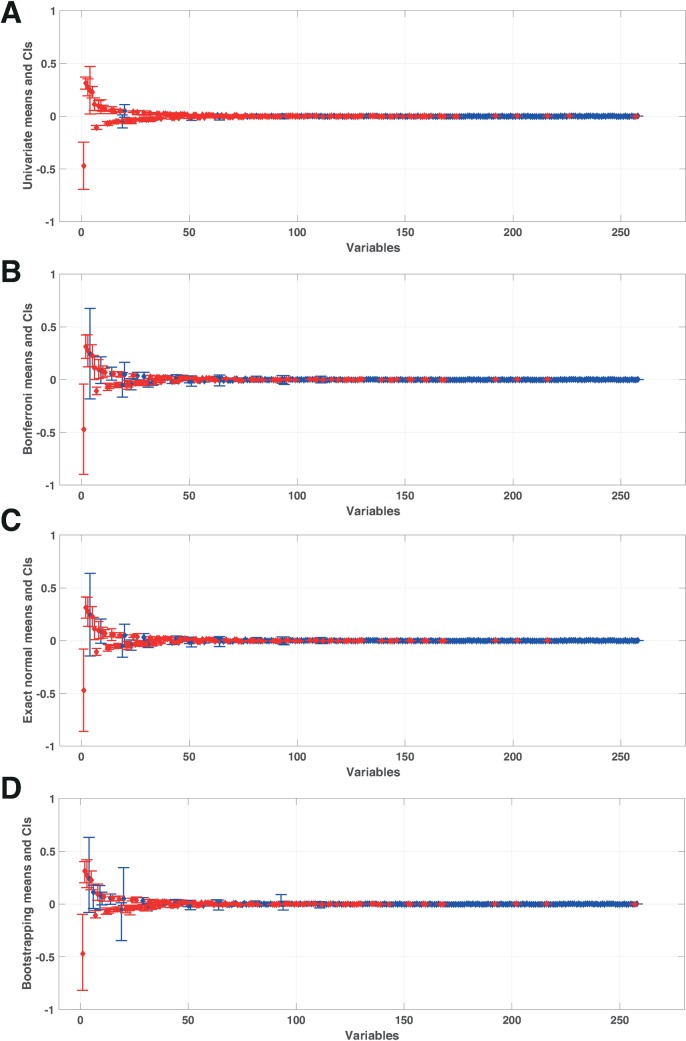
Control coefficients for glucose transport (GLCptspp) and their CIs derived using different methodologies. The diamonds indicate the mean of the FCCs in decreasing order of absolute mean. CIs were derived using (A) univariate t-distributions for *n* = 1 degrees of freedom, (B) Bonferroni, (C) exact normal, and (D) bootstrapping approaches (for more information, see Materials and Methods). The lower and upper whiskers correspond to the CI range. The CIs are blue when they include zero at the 95% confidence level and red if they do not. Numerical values are given in the supplementary information (S2 Table).

The width of a CI is a measure of the uncertainty in a given variable. A wider CI implies a larger range of numerical values that the variable could take within a defined level of confidence. Differences in CIs for the same set of variables constructed using different statistical methods could provide conclusions/recommendations that are method dependent. It is therefore important to know which methodology to apply for a given study. We considered four methodologies for building CIs, which are presented in the Materials and Methods; one without correction, and three that account for the simultaneous coverage level. We used Case 1 as an example to study and compare the CIs. We pre-processed Case 1 data by removing variables that had a standard deviation below a tolerance level of 10^−9^. For insight into which variables have the largest population mean *μ*, Fig 2 displays sample means sorted by absolute value along with their CIs as generated using the four different methods. The method without correction, referred to as the univariate approach, constructs CIs that are considerably narrower (Fig 2A) than the ones constructed using the three multivariate methods that account for simultaneous coverage (Fig 2B, 2C and 2D). These differences in CIs could be interpreted as having a higher confidence in a variable when using the univariate rather than a multivariate approach. Thus, univariate CIs are less accurate for analyzing the system as they take less information into account.

We first applied Bonferroni’s corrections (BCI) for generating the CIs, which are used for increasing the confidence level when testing/comparing multiple variables. We noted that the CI ranges were considerably wider than the ones obtained via univariate t-distributions without correction (Fig 2A and 2B). This was expected as the coverage levels were adjusted for simultaneity and were thus more conservative. The second method applied was the exact normal method (ENCI) that uses correlation of variables to adjust the CIs. The CIs calculated using ENCI were narrower than ones computed with Bonferroni’s correction, as the latter accounts for the dependencies of the variables (Fig 2B and 2C).

**Fig 3:**
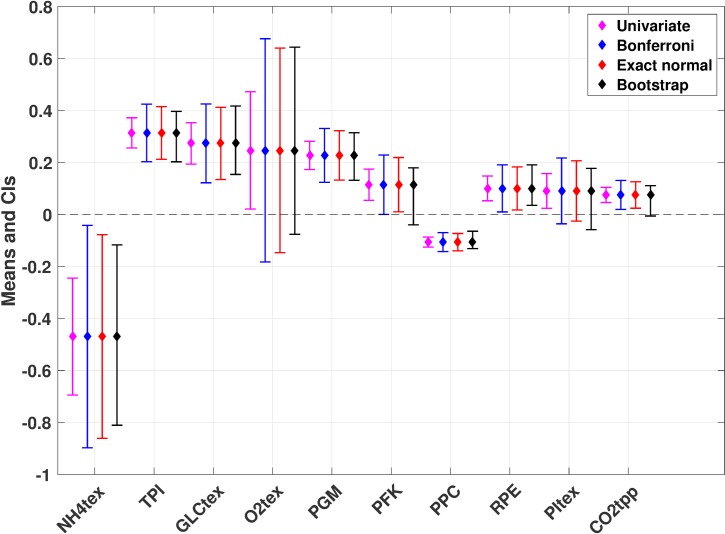
Top flux control coefficients of glucose uptake (GLCptspp) with confidence intervals determined using four different statistical approaches. The top 10 FCCs based on absolute mean are reported with diamonds. The whiskers indicate the CIs for univariate (magenta), Bonferroni (blue), exact normal (red), and bootstrapping (black). The reader is referred to the Materials and Methods for technical details on CI computation. Numerical values are given in the supplementary information (S2 Table).

The third method that we applied for generating CIs is called bootstrapping (BootCIs), which is used when considering non-normal distributions of variables. BootCIs generally produced slightly narrower CIs than the ones derived using ENCI (Fig 2C and 2D). This was also expected, as the BootCIs are less conservative than the exact normal ones. Nevertheless, when the distribution was heavily skewed, the asymmetric CI could be considerably larger on one side of the data point. Asymmetric CIs, such as the BootCIs, do not assume a normal distribution (i.e. symmetry) of the data like with the BCI and the ENCI. Instead, asymmetric CIs account for a systemic bias of the data in a given direction. Hence, the BCI and the ENCI methods are inaccurate when representing asymmetric data and that BootCIs should always be used instead. This happened with the oxygen transport (O2tex), phosphofructokinase (PFK), phosphate transport (PItex), and carbon dioxide transport (CO2tpp) pathways (Fig 3). As BootCIs use the observed data to derive the CIs, they appear more representative and adapted to the studied data.

The computational times for obtaining BCIs, ENCIs, and BootCIs were recorded as 0.12 s, 0.47 s, and 3,520 s, respectively (Mac Pro, 2.7 GHz 12-Core Intel Xeon E5, 64 GB 1866 MHz DDR3 ECC). As expected, the computation was considerably longer for the BootCIs than for both the Bonferroni’s and exact normal methods due to its intense re-sampling (see Materials and Methods).

Even though it is challenging to rank variables based on their uncertainty, with CIs we can still quantify how certain we are about the numerical value of variables. For instance, uncertainty can be used as a way to exclude/select certain variables for further study when designing an experiment. However, differences in the CIs around FCCs could lead to opposing recommendations about which enzymes to target for a given study. For example, if we only considered univariate CIs, we could conclude for Case 1 that PFK is an interesting target enzyme (Fig 3) as it has control over the flux of interest and a relatively narrow CI. However, if we apply the three multivariate methods to construct CIs, we note that these CIs are considerably larger and even include zero within the range for BootCIs, making PFK a less attractive target (Fig 3). Consequently we could make different conclusions about the confidence levels depending on the statistical method, particularly when comparing univariate CIs to ones constructed with a multivariate approach.

Due to the underdetermined nature of the system, alternative steady-state solutions could describe the experimentally observed *E*. *coli* physiology [12]. As mentioned previously, Hameri et al. described four FDPs, herein called cases, and we next considered populations of kinetic models around these four cases and compared their MCA outputs. Studying these outputs can help elucidate why steady-state solutions affect the MCA-based control patterns and metabolic engineering decisions. Fortunately, the three multivariate statistical techniques presented in this publication could be applied for building simultaneous CIs to provide the average difference between two data sets for our case study (see also S1 Methods). These three multivariate techniques were then compared with the often-used univariate approach.

### Case study: Mean difference confidence intervals

We were interested in studying the four operational configurations, FDPs, that could characterize the physiology of aerobically grown *E*. *coli*, which were published by Hameri et al. [12]. We studied their populations of 50,000 stable kinetic models for these four operational configurations. The first was herein labeled Case 1 and was presented in the previous section to demonstrate the different methods for constructing CIs. The three other cases will be referred to as Case 2, Case 3, and Case 4. Cases 2–4 correspond to FDP2-4 from the same publication [12], and we will not discuss their biological differences in this work as the focus lies instead in the CIs. We were interested herein in comparing how different the FCCs for GLCptspp were for these four cases and how conclusions could differ when using different statistical methodologies.

In order to make this case study more comprehensible, we made a pre-selection of the FCCs for GLCptspp that we wanted to compare. For each case, we built the bootstrapped simultaneous CIs and kept the seven FCCs with the largest absolute value in mean amongst those significantly different from zero. The union of these top seven FCCs of the cases was selected for the comparisons, resulting in 15 FCCs to be compared. Since we wanted to compare these 15 FCCs between all four cases, i.e. six possible combinations of two, this resulted in 90 comparisons overall (15 x 6).

**Fig 4:**
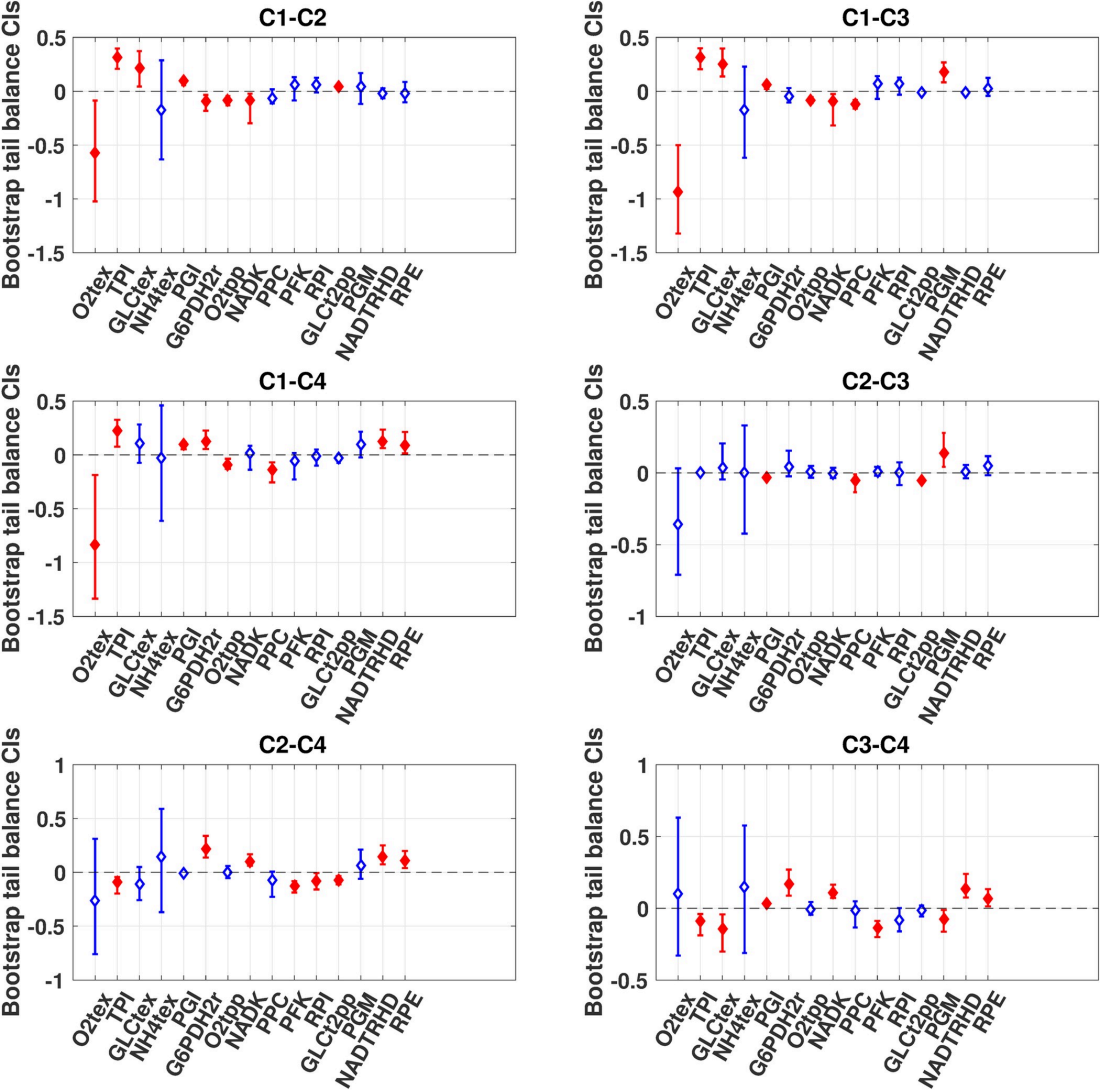
Case study: Differences in means using bootstrapping. Comparison of the differences in means of 15 FCCs for GLCptspp across four cases using the bootstrapping method (see Materials and Methods). The whiskers indicate the CIs, and the diamonds report the estimates of the differences in means. The CIs are blue with a hollow diamond when they include zero at the 95% confidence level and red with a plain diamond if they do not. The tests were carried out globally on the 90 estimates, even though we report each case comparison as a separate plot. Numerical values are given in the supplementary information (S3 Table).

The three multivariate statistical methods presented in Materials and Methods were used to construct CIs for these 90 comparisons. Again, the bootstrapping approach was expected to be the most appropriate because of the aforementioned skewedness of the data. Overall, as shown in Fig 4, 45 comparisons were significant based on the bootstrapping approach. In comparison, the BCI and the ENCI resulted in 44 and 39 significant comparisons, respectively (S1 and S2 Figs). Since the variables have little correlation in this case study, the complexity of the ENCI over BCI seems not to be needed.

We also noted that the comparison of nicotinamide adenine dinucleotide kinase (NADK) between Cases 1 and 2 and between Cases 1 and 3 appeared to be significant when using the BootCIs, whereas the other two multivariate approaches would suggest it is insignificant. This example shows that the BCI and ENCI approaches relying on the normality assumption may lead to different conclusions than the bootstrapping method when dealing with skewed distributions. Comparisons of the other pairs of cases did not reveal notable differences. Overall, there were no major differences in the widths of the CIs derived for the 90 comparisons for these three multivariate statistical methods.

Regarding the overall results in the applications, we observed very few differences between the statistical methods. This led us to prefer BCIs due to their simplicity. The absence of any major differences between these methods can be explained by the fact that the correction for simultaneity is the first and most important aspect before accounting for the dependence and for the skewness of the distribution. This was probably due to the very large number of variables/comparisons in our considered examples. In addition, it was evident that the main factor driving the size of the CIs was the standard deviation of the distributions. We had a clear example of variance inhomogeneity between the variables. If all these techniques take this inhomogeneity reasonably well into account, it is not surprising to see that most variation from one CI to another is indeed due to the standard deviation. This was probably why taking one technique or another did not noticeably change the practical results.

It should also be mentioned that the BCI, even in its simplicity, allows a sample size calculation to estimate the number of samples required to achieve a certain level of confidence (see Materials and Methods, Section 5). This is an a posteriori calculation that is done based on the samples that we already have. For instance, to attain BCIs that have a maximal margin of error of 0.1, we would require around 1.9 million samples for our case studies based on the 90 comparisons that we performed here. Obviously, this estimated number of required samples is subject to the basic and conservative assumptions of the BCI and only serves as an indication.

Additionally, we noted that BootCIs provided certain minor advantages over the other methods at the cost of a higher computational effort, particularly when the distributions were heavily tailed or asymmetric. Hence, if the additional computational resources are available, it might be safer to apply the BootCIs when dealing with these kinds of data sets. As this was clearly the case for us and we had the computational resources, it was certainly worth investigating and applying the BootCIs to obtain our results. Should the BootCI approach be too complex to implement computationally, it would be worthwhile to consider the ENCI over the BCI in the presence of high dependencies between the variables because the ENCI accounts for the correlation of the variables.

**Fig 5:**
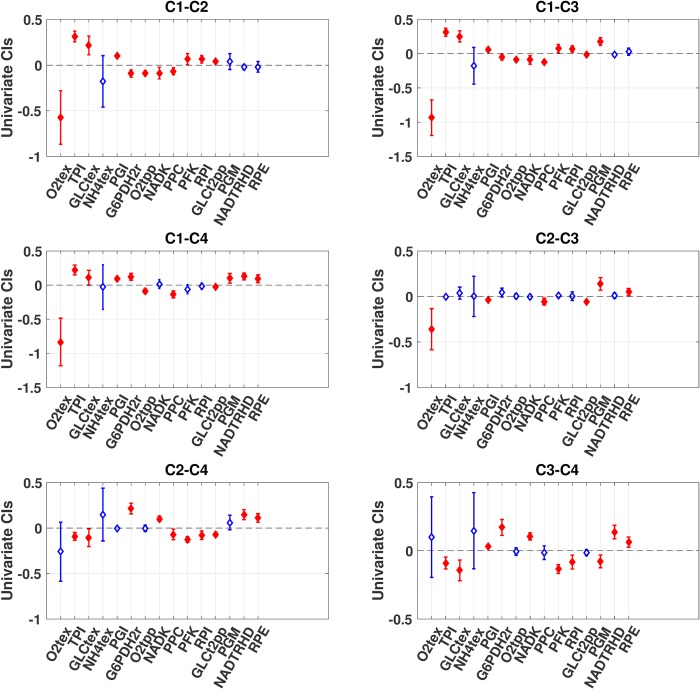
Case study: Differences of means using the univariate method. Comparison of the differences in means of 15 FCCs for GLCptspp across four cases using the univariate method (see Materials and Methods). The whiskers indicate the CIs, and the diamonds report the estimates of the differences in means. The CIs are blue with a hollow diamond when they include zero at 95% confidence level and red with a plain diamond if they do not. The tests were carried out globally on the 90 estimates even though we report each case comparison as a separate plot. Numerical values are given in the supplementary information (S3 Table).

The most distinct observation comes again when we contrast these three multivariate approaches (Figs 4 and S1 and S2) to the univariate one (Fig 5). The univariate method would suggest that 60 comparisons out of the 90 are actually significant. This is considerably more than the multivariate approaches, which had at maximum 45 significant comparisons, or the bootstrapping case (Fig 4). Using the univariate approach could result in claiming that more differences between the model outputs are significant when they are actually not according to the multivariate approaches. Thus, multivariate methods should be favored when making model-based decisions based on multiple variables rather than applying the standard univariate approach.

## Conclusion

We hereby introduced, to our knowledge, the first computational workflow for assigning statistical significance to EM outputs in systems biology. This work studied how alternative statistical approaches could be applied for computing CIs for the MCA outputs of populations of non-linear kinetic models of the metabolism of aerobically grown *E*. *coli*. We investigated the differences in three distinct methods—Bonferroni’s correction, exact normal, and bootstrap—in calculating simultaneous CIs and discussed the particularities and assumptions of these approaches. We demonstrated that we could successfully use these three methods to build CIs for populations of models and that there were no considerable differences in the CIs derived for the presented data using the three methods. Therefore, Bonferroni’s correction was remarkable for its simplicity and for its ability to estimate the sample sizes required for achieving a certain confidence level. We highlighted that the bootstrapping approach, although more complicated computationally and algorithmically, provided certain clear advantages when handling data with highly asymmetric and/or skewed distributions. Independent of the method used, it was crucial to consider the multivariate correction of CIs for their simultaneity. Univariate CIs could lead to misleading model-based conclusions when comparing populations of multiple variables. Hence, we propose a workflow (Fig 1) that can be used to construct CIs for the outputs—not only limited to control coefficients derived from MCA—of populations of kinetic models.

## Materials and methods

### Kinetic model

A published kinetic model [12] of aerobically grown *E*. *coli* was used for the purpose of this study. In the original publication, the authors presented four different operational configurations referred to as flux directionality profiles (FDPs). The populations of 50,000 kinetic models for each FDP were considered herein for comparing the distributions of flux control coefficients (FCC) with the presented statistical methods. For further information about the populations of kinetic models used here, we refer the reader to the original publication [12].

### Simultaneous CIs for variable significance

A CI is an interval that contains the population mean *μ* with a probability of 1 – *α*. This probability is also known as the coverage. The population mean can be thought of as the limit sample mean as *n* tends to infinity. The CI is built from the sampled data and is thus random. The coverage is to be understood as the proportion of times the CI would contain *μ* if the sampling were repeated a large number of times.

The variable significance is judged by its population mean *μ* estimated by the sample average. This estimate is tainted by uncertainty due to data variation, and this uncertainty is quantified by CIs. Because of the equivalence between a statistical test and CI, to be of real importance, a variable sample average should be large in absolute with a CI bounded away from zero to ensure that this large estimated value is due to pure chance. In the following, several constructions of CI are presented.

#### Univariate and simultaneous CIs

CIs can be built using numerous techniques and for any variables. The most well-known CIs for the mean are univariate and based on the t-distribution. To account for the variability, univariate CIs at level of 1 – *α*, for *α* = 5%, are added around each sample mean (see Fig 2). Checking that the CI contains 0 is equivalent to making a statistical test that *μ* = 0 at level *α*. All technical details are included in the Supplementary materials (S1 Methods).

Used as such, univariate CI are misleading, since a correction for the inspection of *p* variables is needed. This need, well-known for multiple testing [16], is the same for CIs. Indeed, the simultaneous coverage of several CIs, which is the probability of containing all population means, may be much lower than each univariate coverage. In the remainder of this section, we present three ways to build a corrected CI, called a simultaneous CI.

#### Bonferroni’s simultaneous confidence interval (BCI)

The Bonferroni’s correction, probably the most used method, guarantees the simultaneous coverage 1 – *α*_*S*_ by dividing the univariate *α* levels by *p*, giving *α* = *α*_*S*_ / *p*, where *α*_*S*_ is defined as the simultaneous confidence level of multiple CIs. For example, with two variables, each CI is built at *α* = .025 and the simultaneous coverage is (1−*α*)^2^ = .975^2^ = .951. On a larger scale, such as with our 275 variables, the simultaneous coverage without correction would be .95^275^ ≈ .00 if the variables were all independent. Hence, it is almost certain that at least one of the population means is not contained in the corresponding CI. This correction is approximate and correct only if the variables are all independent (see S1 Methods). Otherwise, it is often too conservative, which means that the CIs are too wide [17].

An important aspect is that, even when Bonferroni’s correction (or any other) is appropriate, the univariate coverage will be 1 – *α* > 1 – *α*_S_. Thus, taken individually, each CI is conservative. This is the cost of having simultaneous correction and is illustrated in Fig 2B. The independence assumption of Bonferroni’s correction is not often satisfied, as shown in our case in (S4 Table). Hence, we felt encouraged to consider alternative approaches that account for the dependency of the variables being compared.

#### The exact normal (ENCI)

The exact normal method [18] attempts to release Bonferroni’s assumption of independence between the CIs. The method uses multivariate normal distributions *N*_*p*_(0, *Γ*) to correct for the dependencies of the *p* variables using an estimate of *Γ*, the correlation matrix of the observations. If the variables exhibit dependence, the resulting simultaneous CIs are expected to be smaller than the ones derived with Bonferroni’s correction. The price to pay is in terms of computational and mathematical complexity. For the technical details, see Supplementary materials (S1 Methods).

Both the exact normal and the Bonferroni’s correction rely on the normal distributions assumption for constructing CIs. However, extreme observations and asymmetry in the data justify using methods that relax this assumption.

#### Bootstrapped simultaneous CI (BootCI)

Originated from Beran’s work [19], the BootCIs generalize the exact normal by relaxing the normality assumption. The approach is based on the data re-sampling to estimate a root statistical distribution. Coupled with a pre-pivoting technique, tail balancing, and under some technical assumptions, the method provides asymmetric simultaneous CIs with

- the correct target simultaneous coverage
- equal marginal coverages
- outside tail balance, i.e. the same probability on both sides out of the CIs.

The assumption for the bootstrap method is less strict than that for the normality method in the sense that it does not assume distribution symmetry, but it must be valid. Unfortunately, it cannot be validated in practice and remains an a priori assumption. Nevertheless, the data analyzed in this study was significantly skewed (S3 Fig), which suggested the appropriateness of using bootstrapping.

To the best of our knowledge, BootCIs are the most generally available method that can be used without any further assumption. They are always more correct than Bonferroni’s and exact normal CIs in that if the assumptions of those methods are valid, the one of the bootstrapped method is also valid. The price to pay for the bootstrapping method is yet another level of technicality and computational complexity. For the technical details, see Supplementary materials (S1 Methods).

### Confidence intervals for comparing cases

The comparison of two cases is made by building CIs based on the difference of their means. When cases are compared along several variables, simultaneous CIs must be used and can be built using the three methods highlighted in the previous section. In applications, simultaneous CIs are used for multiple comparisons (for example see Miller et al. [20] for a detailed treatment). Because of the correction for simultaneity, the variables along which the cases differ can be tested: the differences are significant whenever zero does not belong to the interval (see Fig 2 for the application). The mathematical details are reported in the Supplementary materials (S1 Methods).

### Sample size calculation

The uses of confidence intervals and of power analysis are well known in computing required sample sizes (for example see Goodman et al. [21] for a good overview in the context of clinical research). The general concept is that the width of a CI is reduced when the sample size increases. Since this width describes the uncertainty of the corresponding mean, the sample size required to achieve a given width can be computed.

However, the sample size computation requires prior knowledge or prior data gathering. Indeed, the length of the CI also depends on the standard deviation that must be guessed or estimated beforehand. In our application, we thus made an a posteriori sample size calculation based on the estimated standard deviation from the available sample. We also used BCI because it is the only method that allows an explicit sample size formula (see Supplementary materials [S1 Methods] for the technical details). Even if approximate, this calculation would be quite demanding with the other methods, if not intractable for the bootstrapping approach.

## Supporting information

S1 Methods Algorithms and further details for deriving confidence intervals. (DOCX)

S1 Table Reaction and metabolite names for the model. (XLSX)

S2 Table Computed means and confidence intervals for Case 1. (XLSX)

S3 Table Computed means and confidence intervals for Case 1–4 comparisons. (XLSX)

S4 Table Correlation matrix for Case 1 variables. (XLSX)

S1 Fig Case study: Differences of means using the Bonferroni method. Comparison of the differences in means of 15 FCCs for GLCptspp across four cases using the Bonferroni method (see Materials and Methods). The whiskers indicate the CIs and the diamonds report the estimates of the differences in means. The CIs are blue with a hollow diamond when they include zero at the 95% confidence level and red with a plain diamond if they do not. The tests were carried out globally on the 90 estimates, even though we report each case comparison as a separate plot.
(EPS)

S2 Fig Case study: Differences of means using the exact normal method. Comparison of the differences in means of 15 FCCs for GLCptspp across four cases using the exact normal method (see Materials and Methods). The whiskers indicate the CIs and the diamonds report the estimates of the differences in means. The CIs are blue with a hollow diamond when they include zero at the 95% confidence level and red with a plain diamond if they do not. The tests were carried out globally on the 90 estimates, even though we report each case comparison as a separate plot.
(EPS)

S3 Fig Distributions of control coefficients highlighting non-normal nature of the data. FCC distributions for the control of GLCptspp with respect to (A) ribulose 5-phosphate 3-epimerase (RPE), (B) PFK, and (C) triose-phosphate isomerase (TPI).
(EPS)
