# Supplementary material for: Statistical inference in ensemble modeling of cellular metabolism
Source: PLoS Comput Biol. 2019 Dec 9;15(12):e1007536. doi: 10.1371/journal.pcbi.1007536 (PMC6922442; doi:10.1371/journal.pcbi.1007536)
Supplement: S1 Methods — (DOCX) [file pcbi.1007536.s001.docx]

# Supporting Information

# Hameri et al.

# S1 Methods

# Supplementary notes on statistical methods used

## Confidence intervals

### Univariate CI

For an independent sample $x_{1},\ldots,x_{n}$, the CI is built from the mathematical result that, under very general conditions, if $\mu$ is the true population mean, then the statistic $T=(\bar{x}-\mu)/(n^{-1/2}s)$ follows a Student t-distribution with $n-1$ degrees of freedom. Here, $\bar{x}$ and $s$ denote the sample mean and standard deviation. Denoting $t_{\beta}$ the $\beta$-quantile of the t-distribution with $n-1$ degrees of freedom, for any $0<\beta<1$, it follows that, for any level $0<\alpha<1$, $P\left(|T|\leq t_{1-\alpha/2}\right)=1-\alpha$. Reversing the inner term, one obtains $P\left(\bar{x}-n^{-1/2}st_{1-\alpha/2}\leq\mu\leq\bar{x}+n^{-1/2}st_{1-\alpha/2}\right)=1-\alpha$. The inner bounds form the confidence interval at level $1-\alpha$, which is the coverage, that is, the probability that the CI contains the population mean $\mu$.

### BCI

The Bonferroni’s correction mathematically defines the CIs of variable j as:

$$C_{j}=\left[\bar{x}_{j}-n^{-1/2}s_{j}t_{1-\alpha/2p},\bar{x}_{j}+n^{-1/2}s_{j}t_{1-\alpha/2p}\right]$$

where *p* is the number of variables, *n* is the number of observations*,* $1-\alpha$ is the simultaneous coverage*,* $\bar{x}_{j}$ is the sample mean, $s_{j}$ is the standard deviation of variable $x_{j}$.

To justify this correction, suppose we have two variables to study, and thus two univariate CIs, $C_{1}$ and $C_{2}$, built to estimate their true means $\mu_{1}$ and $\mu_{2}$, both coverage of $1-\alpha=.95$. Assume further that they are independent and have the correct coverages. Then, without correction, the simultaneous coverage would be . This is below the target of .95. With the correction, the univariate correction would be $1-\alpha/2=0.975$ and the simultaneous coverage would be $.975^{2}\approx.951$, very close to the target.

### ENCI

The exact normal method developed by Miller [[1](#_ENREF_1)] attempts to release the assumption of independence of the Bonferroni’s correction. The exact normal method defines the CIs as $C_{j}=\left[\bar{x}_{j}-n^{-1/2}s_{j}q_{1-\alpha},\bar{x}_{j}+n^{-1/2}s_{j}q_{1-\alpha}\right]$, where $q_{\beta}$ is the $\beta$-quantile of the distribution of $\max_{j=1,\ldots,p}\left|z_{j}\right|$, for $z=(z_{1},\ldots,z_{p})^{T}$ multivariate normal $N_{p}(0,\Gamma)$. The variance matrix $\Gamma$ is the correlation matrix of the observations and can be estimated. To find $q_{1-\alpha}$ such that the simultaneous coverage is equal to $1-\alpha$, the following algorithm can be used:

1. For $r=1,\ldots,R$,
   1. Simulate $(z_{1}^{(r)},\ldots,z_{p}^{(r)})$ from $N_{p}(0,\Gamma)$
   2. Compute $m^{(r)}=\max\{|z_{1}^{(r)}|,|z_{2}^{(r)}|,\ldots,|z_{p}^{(r)}|\}$
2. Estimate $q_{1-\alpha}$, the $(1-\alpha)$-quantile of $m^{(1)},\ldots,m^{(R)}$.
3. Set $C_{j}=\left[\bar{x}_{j}-n^{-1/2}s_{j}q_{1-\alpha},\bar{x}_{j}+n^{-1/2}s_{j}q_{1-\alpha}\right]$, for $j=1,\ldots,p$.

The number of Monte Carlo simulations $R$ should be large enough to estimate the quantile. Typically, for $\alpha=.05$, $R=1000$ provides 50 points to estimate $q_{1-\alpha}$, which is considered as large enough. The marginal coverage of each $C_{j}$ is approximately $1-\beta$, where $\beta$ is such that $q_{1-\alpha}=t_{1-\beta/2}$.

### BootCI

The bootstrapping method detailed below goes back to Beran in [[2](#_ENREF_2)]. The method releases the normality assumption by using a bootstrap estimate. In particular, the resulting CIs are not necessarily symmetric. Beran used a pre-pivoting technique to guarantee equal marginal coverage: . We further added a tail balancing condition guaranteeing that each side outside the CIs has the same probability. Let us write , then the tail balancing guarantees that . We are not aware of the application of this last technique, even if it is quite straightforward and similar approaches were used (see [[3](#_ENREF_3)] for treating the unbalanced design case).

Following the notation of Beran [[2](#_ENREF_2)], each univariate CI, , where $t_{j}=(\bar{x}_{j}-\mu_{j})/(n^{-1/2}s_{j})$. Assuming that $t_{j}$ is a pivot means that its distribution $H_{j}$ is independent of $\mu_{j}$ (and $\sigma_{j}$). Therefore, . Since we want balanced CIs and tail balancing, we fix , where $1-\beta$ is the univariate coverage of each $C_{j}$. These $C_{j}$ can be then written as

$$C_{j}=\{\mu_{j}:|H_{j}(t_{j})-1/2|\leq(1-\beta)/2\}$$

The simultaneous CI is thus $C=\{\mu:\max_{j=1,\ldots,p}|H_{j}(t_{j})-1/2|\leq(1-\beta)/2\}$. Now, fixing the simultaneous coverage at $1-\alpha$, we have that $(1-\beta)/2$ is the $(1-\alpha)$-quantile of $H$, the distribution of $\max_{j=1,\ldots,p}|H_{j}(t_{j})-1/2|$. Using the bootstrap, we can simulate the distributions $H$ and $H_{j}$, and compute $\beta$, and $u_{j}$. The algorithm is

1. For $r=1,\ldots,R$,
   1. Draw with replacement $x_{1}^{(r)},\ldots,x_{p}^{(r)}$ from the data set $x_{1},\ldots,x_{p}$.
   2. For $j=1,\ldots,p$, compute $t_{j}^{(r)}=|\bar{x}_{j}^{(r)}-\bar{x}_{j}|/(n^{-1/2}s_{j}^{(r)})$, where $s_{j}^{(r)}$ is the standard deviation of $x_{1j}^{(r)},\ldots,x_{nj}^{(r)}$.
2. Compute $h_{j}^{(r)}=(\text{rank}\{t_{j}^{(r)}\text{ in }t_{j}^{(1)},\ldots,t_{j}^{(R)}\}-1)/(R+1)$,
3. Compute $h_{r}=\max_{j=1,\ldots,p} |h_{j}^{(r)}-1/2|$.
4. Compute $q_{1-\alpha}$, the $(1-\alpha)$-quantile of $h_{1},\ldots,h_{R}$ and set $\beta=1-2q_{1-\alpha}$
5. For each$j=1,\ldots,p$, compute and $u_{j}$ the quantiles at levels $\beta/2$ and $1-\beta/2$ of $h_{j}^{(1)},\ldots,h_{j}^{(R)}$.
6. For each$j=1,\ldots,p$, set .

The number of Monte Carlo repetition $R$should be large enough to provide a good estimate of and $u_{j}$. Since $\beta$ is typically very small, this number can be very large. For example, with $\beta/2=.0001$ then $R=10/.0001=10'000$ will provide only 10 points to estimate and $u_{j}$. This method is based on the assumption that the so-called root statistics $t_{j}=(\bar{x}_{j}-\mu_{j})/(n^{-1/2}s_{j})$ is a pivot, that is a random variable whose distribution is independent of the population parameters $\mu_{j}$ and $s_{j}$. If this assumption is already considerably lighter than the normal one, it still is needed for the bootstrap to be valid. Unfortunately, this assumption cannot be checked in practice.

## Simultaneous confidence intervals for comparisons of means

We can build simultaneous confidence intervals for the difference between two cases and make pairwise comparisons. To do so it suffices to adapt the root statistic to mean differences $t_{abj}=(\bar{x}_{aj}-\bar{x}_{bj})/(n^{-1/2}s_{abj})$. There $a$ and $b$ denote the two cases, $j$ denotes the variable, and $s_{abj}$ is the pooled standard deviation $s_{abj}=\{(s_{aj}^{2}+s_{bj}^{2})/2\}^{1/2}$. Here it is assumed that the design is balanced.

## Sample size calculations

CIs can readily be used for computing the estimated number of samples necessary in order to reach a required length for a given level of confidence (see [[4](#_ENREF_4)] for a good overview). The principle is to fix the margin of error (MoE) which is the half-length of the CI. When applied to BCIs, it is $MoE_{j}=n^{-1/2}s_{j}t_{1-\alpha/(2p)}$. We can reorder the above equation to solve for $n_{j}$ to obtain the number of samples required to achieve a given $MoE_{j}$, $n_{j}=(s_{j}t_{1-\alpha/(2p)}/MoE_{j})^{2}$. The target $MoE_{j}$ has to be fixed and $s_{j}$ should be estimated with prior data. The required number of samples will be selected as the maximum of all $n_{j}$. The result remains an estimate and it could be considerably different in reality.

# References SI

1. Rupert Jr G. Simultaneous statistical inference: Springer Science & Business Media; 2012.

2. Beran R. Balanced simultaneous confidence sets. Journal of the American Statistical Association. 1988;83(403):679-86.

3. Tu W, Zhou X-H. Pairwise comparisons of the means of skewed data. Journal of Statistical Planning and Inference. 2000;88(1):59-74.

4. Goodman SN, Berlin JA. The use of predicted confidence intervals when planning experiments and the misuse of power when interpreting results. Annals of internal medicine. 1994;121(3):200-6.
